# Examining the Utility of Rapid Salivary C-Reactive Protein as a Predictor for Neonatal Sepsis: An Analytical Cross-Sectional Pilot Study

**DOI:** 10.3390/diagnostics13050867

**Published:** 2023-02-24

**Authors:** Chaitra Ramavath, Shravan Kumar Katam, Venkateshwarlu Vardhelli, Saikiran Deshabhotla, Tejo Pratap Oleti

**Affiliations:** Department of Neonatology, Fernandez Foundation, Fernandez Hospital, Unit-2, Opp. Old MLA Quarters, Hyderabad 500029, India

**Keywords:** neonatal sepsis, C-reactive protein, CRP, salivary CRP

## Abstract

This study aimed to compare the rapid bedside quantitative assessment of C-reactive protein (CRP) in saliva to serum CRP to predict blood culture-positive sepsis in neonates. The research was carried out over eight months at Fernandez Hospital in India (February 2021–September 2021). The study included 74 randomly selected neonates with clinical symptoms or risk factors of neonatal sepsis requiring blood culture evaluation. SpotSense rapid CRP test was conducted to estimate salivary CRP. In analysis, the area under the curve (AUC) on the receiver operating characteristics (ROC) curve was used. The study population’s mean gestational age and median birth weight were 34.1 weeks (SD: ±4.8) and 2370 g (IQR: 1067–3182). The AUC on ROC curve analysis for predicting culture-positive sepsis was 0.72 (95% CI: 0.58 to 0.86, *p*-value: 0.002) for serum CRP and 0.83 (95% CI: 0.70 to 0.97, *p*-value: <0.0001) for salivary CRP. The Pearson correlation coefficient between salivary and serum CRP was moderate (r = 0.352, *p*-value: 0.002). Salivary CRP cut-off scores were comparable to serum CRP in terms of sensitivity, specificity, PPV, NPV, and accuracy in predicting culture-positive sepsis. The rapid bedside assessment of salivary CRP appears to be an easy and promising non-invasive tool in culture-positive sepsis prediction.

## 1. Introduction

Neonatal sepsis is one of the leading causes of morbidity and mortality in neonates worldwide and in India [1,2], affecting approximately three million babies annually, with a mortality rate ranging from 9% to 20% in severe sepsis [3]. This high mortality rate is most likely the result of delays in adequate diagnosis and treatment. Blood culture is still considered as the ‘gold standard’ diagnostic test for sepsis. However, culture findings can take up to 48 h, and overreliance on them might result in unnecessary delays in treatment and severe complications, including death. Serum C-reactive protein (CRP) is an acute-phase reactant that is commonly used in conjunction with other parameters in a sepsis screen [4,5,6]. Serum CRP assays, whether performed alone or in conjunction with other relevant tests and biomarkers, play an essential role in guiding treatment decisions in neonates with suspected sepsis before receiving blood culture results [7,8]. However, CRP and other biomarkers currently rely on serum or whole blood samples, necessitating blood sampling and laboratory support. Early administration of antimicrobials is the most effective way to decrease the mortality and morbidity related to sepsis. The Surviving Sepsis Campaign guidelines for the management of sepsis and septic shock advocate administering the antibiotic within 1 h of the suspicion of sepsis [9]. It has been shown that early antibiotic administration improved neonatal sepsis outcomes [10]. Serum CRP processing may take hours as it needs laboratory support, and it also requires blood sampling, which can lead to more phlebotomy losses and pain in neonates. Decreasing the number of procedures is one of the effective interventions to decrease pain in neonates [11]. Especially in very preterm or very low birth weight neonates, frequent blood sampling can lead to more blood transfusions. Using non-invasive techniques for investigations can help in decreasing the pain and blood transfusion requirements due to invasive procedures [12]. It is also very helpful to monitor serially to understand the response to therapy or disease progression. A novel, rapid, non-invasive point-of-care device which requires less technical expertise to test CRP would be an ideal alternative to serum CRP in diagnosing sepsis.

CRP is preferentially transported toward the direction of saliva, highlighting the possibility of using this molecule as a salivary marker [13]. Several earlier researchers have found a positive correlation between salivary and serum CRP, advocating using salivary CRP as a screening tool for neonatal sepsis. However, there were concerns regarding sample collection techniques and sample volume constraints and the processing of the test. Previous studies used batch-processing lab methods such as ELISA, which necessitate sample storage and pre-processing [14,15,16,17,18,19]. These processing methods require expert personnel and laboratory support leading to a time lag in diagnosis and treatment. Moreover, these studies have focused on the correlation with serum CRP but not focused on the ability to predict culture-positive sepsis. The novel Spotsense salivary CRP test by lateral flow assay is a rapid, non-invasive bedside quantitative test that requires a very minimal salivary sample which can be obtained easily. It needs less technical expertise, does not require any sample processing, and gives the results immediately. This study aimed to test the efficacy and performance of a novel rapid bedside test for the quantitative estimation of salivary CRP by lateral flow assay in newborns with suspected sepsis, bypassing these previous limitations.

## 2. Materials and Methods

This analytical cross-sectional pilot study was carried out at the tertiary care NICU in Fernandez Hospital, Hyderabad, Telangana, India from February 2021 to September 2021. Prior approval from the Institute’s research ethics committee (IEC) was obtained (Fernandez Hospital IEC_Ref_No: 12_2020). The study included neonates with risk factors and/or who developed clinical symptoms of sepsis and required blood culture after obtaining informed parental consent, randomly, as per the investigating team’s availability at the time of sampling. Neonates with any gestational age and birth weight who were admitted to NICU and requiring blood culture testing before day 28 of life were eligible for the study.

### 2.1. Salivary CRP Diagnostic Kit and Measurement

**Figure 1:**
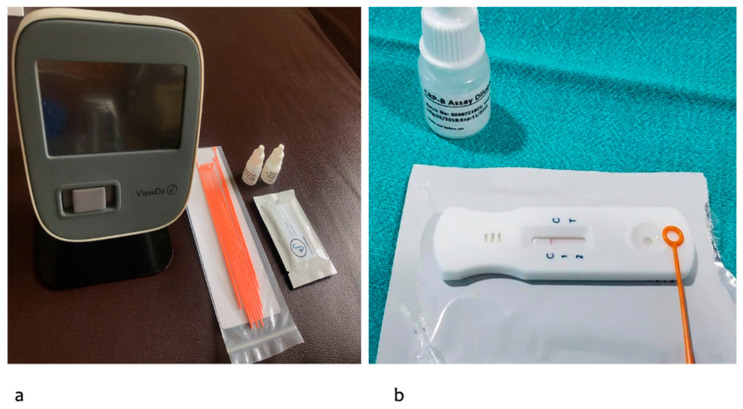
(**a**): SpotSense VIEWDx analyzer along with salivary CRP test kit; (**b**): CRP test cartridge, a sample collector loop, and a desiccant.

For the Salivary CRP, measurements were performed using the SpotSense Salivary CRP kit and a VIEWDx analyzer (Figure 1a). It is an in vitro, rapid, bedside diagnostic colorimetric lateral flow assay designed to quantify CRP in human salivary samples. Each test box has 20 individually packed test kits and one assay diluent tube (1 tube is sufficient to run >20 tests). Each test kit comes with a CRP test cartridge, a sample collector loop, and a desiccant. A sample pad, a conjugate pad with gold nanoparticles coupled with anti-human CRP, a membrane with mouse monoclonal anti-human CRP at the test line, and a rabbit IgG at the control line are all included in the CRP test cartridge. Gamma radiation is used to sterilize and seal each kit. The test is shipped at room temperature and must be stored sealed at 4–30 °C. The sealed pouch should be opened only before the testing. The pouch should be brought to an operating temperature (15–40 °C) before opening, in case stored by refrigeration.

**Figure 2:**
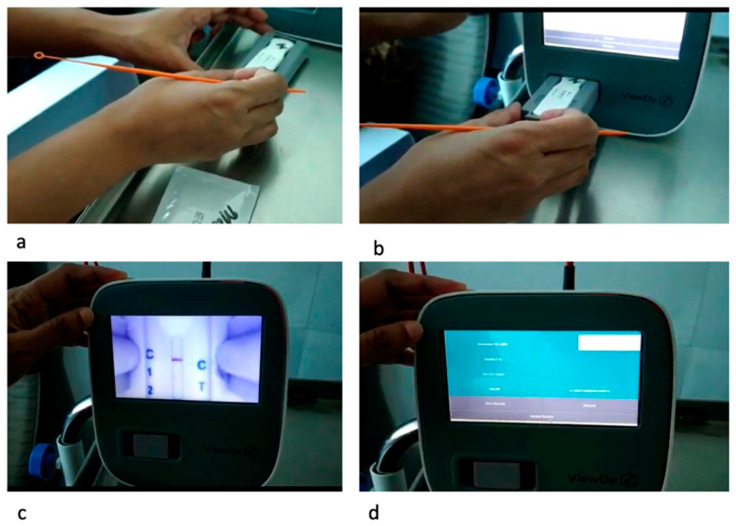
Steps of inserting the test cartridge in the VIEWDx analyzer and running the assay. (**a**): Insert the cartridge into the cartridge port; (**b**): Insert the cartridge and along with the port into the slot on the VIEWDx analyzer; (**c**): Running the assay (**d**): Display of results on the screen.

The SpotSense assay is a rapid test with a 10 min turnaround. Sterile loops are provided with the SpotSense Salivary CRP test to collect the saliva samples. The loop with saliva is placed over the sample well of the test cartridge, and two drops of the assay diluent are added (Figure 1b). It should be ensured that the test is run correctly, and a sharp control line is formed. The kit with a visual interpretation chart should be referred to for any unusual issues with assay kits (Supplementary Material Figure S1). Once the test run is over, the test cartridge is placed in the VIEWDx analyzer, and in-device instructions are followed to obtain the readings (Figure 2). The test should be repeated on a different assay in case of an error. It is recommended to perform a minimum of one quality control test after opening a new test to ensure optimal test performance. Consequently, further quality control tests should be performed at regular intervals.

The limit of detection for salivary CRP was assessed and was found to be 1 ng/mL. The test can assess the maximum value up to 3000 ng/mL. The salivary CRP values below the level of 1 ng/mL will be shown as zero, and the levels above 3000 ng/mL will be shown as >3000 ng/mL. The test is specific to the CRP molecule and does not have any cross-reactivity with other salivary biomolecules such as procalcitonin, bilirubin, cytokines, albumin, and cortisol. The inter-assay coefficient of variability (CV) and intraassay CV were examined before conducting the research. The inter-assay CV was calculated by evaluating the CRP standards at 5 ng/mL (low) and 100 ng/mL (high) on five separate assays each. The overall mean, standard deviation, and CV were calculated individually for both of the normal concentrations. The average %CV at low and high concentrations was reported as inter-assay CV. The %CVs at low and high concentrations and the overall average were 6.2%, 5.4%, and 5.8%, respectively. The intra-assay CV was measured by examining 50 salivary samples in duplicates on the CRP test strips. The %CV for each sample is computed by taking the standard deviation of the sample duplicates’ findings, dividing it by the duplicate mean, and multiplying by 10. The intra-assay CV was calculated by taking the average individual CVs and was found to be 5.2%.

### 2.2. Serum CRP Measurements

Serum CRP measurements were taken using the immunoturbidometric method on the Dimension^®^ RxL Max^®^ Integrated Chemistry System by Siemens. This process requires a sample of 2 mL blood in a plain vacutainer and requires the centrifugation of the blood sample. After the centrifugation, the sample is processed through the C-reactive protein extended range (RCRP) method which is based on the particle-enhanced turbidimetric immunoassay (PETIA) technique. Synthetic particles coated with antibodies to C-reactive protein (AbPR) aggregate in the presence of CRP in the sample. The increase in turbidity which accompanies aggregation is proportional to the CRP concentration. The analytical range is 0.05 mg/dL to 25.00 mg/dL. The usual turnaround time for serum CRP was 2–3 h in our laboratory.

Salivary CRP was measured in infants who needed blood culture testing either during or after serum CRP estimation. To minimize significant variations in CRP levels, salivary CRP samples were collected within 1 h of serum CRP sampling. Salivary CRP samples were obtained at least 30 min after the feed to avoid any contamination with breast milk. The relevant antenatal, perinatal, and postnatal variables of the enrolled neonates were collected.

The culture-positive sepsis was defined as a positive result on one or more bacterial or fungal blood cultures obtained from the blood of a neonate with clinical signs of infection (i.e., temperature instability, irritability, apathy, feeding difficulties, prolonged capillary refill, apnoea, tachycardia, and tachypnoea). Probable sepsis was defined as an episode with clinical signs of infection or with the presence of risk factors for sepsis along with any positive septic screen parameter in the absence of a positive bacterial or fungal culture. The positive septic screen includes any of the following four positive parameters, i.e., serum CRP more than 1 mg/dL, absolute neutrophil count (ANC) less than 1500/mm^3^, total white blood cell count (WBC) less than 4500/mm^3^, and immature to mature neutrophil ratio (IT ratio) of >0.2.

### 2.3. Statistical Analysis

The predictive value of serum and salivary CRP in detecting culture-positive neonatal sepsis was evaluated using receiver operating characteristics (ROC) analyses. The area under the curves (AUCs) on the ROC curve analysis for both approaches were compared. Using the Youden index, the optimal cut-off values were calculated from the ROC curve. Based on the cut-off scores, the sensitivity, specificity, positive predictive value (PPV), negative predictive value (NPV), and accuracy in diagnosing culture-positive sepsis were calculated. Correlations between salivary and serum CRP readings were also calculated for each group. SPSS version 28 (Armonk, NY, USA: IBM Corp) was used for statistical analysis. As it was a pilot study, a convenient sample size of 74 neonates was recruited.

## 3. Results

Table 1 describes the characteristics of the study population. The study population’s mean gestational age and median birth weight were 34.1 (SD: ±4.8) weeks and 2370 (1067–3182) g, respectively. About 56% of the neonates were enrolled within 72 h after birth. The incidence of culture-positive sepsis was 24.3% (*n* = 18), with the proportion of newborns with early onset sepsis being 5.4% (*n* = 3) and late-onset sepsis being 83.3% (*n* = 15). Most organisms grown in blood culture were Gram-negative (15/18: 83%). The predominant Gram-negative organisms included *Klebsiella* sp. (*n* = 8, 44%), *Enterobacter* (*n* = 3, 16.6%), and *E. Coli* (*n* = 2, 11.1%).

**Figure 3:**
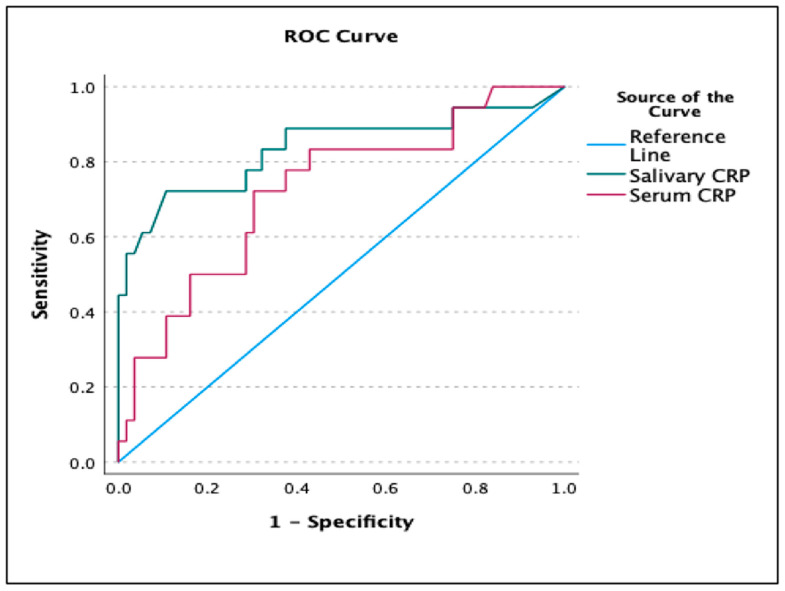
Comparison of AUC between ROC curve of serum CRP and salivary CRP for predicting culture-positive sepsis.

The salivary CRP assay was successful in 94% of the salivary CRP kits used. The median (IQR) serum CRP and salivary CRP values in the overall study population were 1.85 (0.75–4.07) mg/dL and 6.1 (2.19–12) ng/mL, respectively. The median serum and salivary CRP values were significantly different across the three groups (culture-positive sepsis, neonates with probable sepsis, and neonates only with risk factors) (Table 2). The AUCs on ROC Curve analysis for predicting culture-positive sepsis for serum and salivary CRP 0.72 (95% CI: 0.58 to 0.86, *p*-value: 0.002), 0.83 (95% CI: 0.70 to 0.97, *p*-value: <0.0001), respectively (Figure 3).

**Figure 4:**
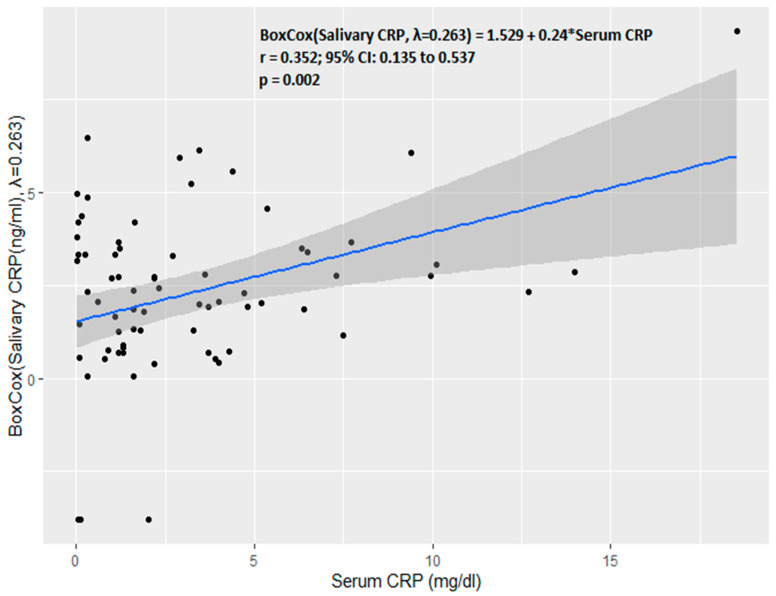
Correlation between serum CRP and salivary CRP.

In the study population, the serum and salivary CRP cut-off scores for predicting culture-positive sepsis were 2.8 mg/dL and 11.6 ng/mL. Table 3 shows the sensitivity, specificity, PPV, NPV, and accuracy of the cut-off scores in predicting culture-positive sepsis for serum and salivary CRP. On the ROC curve, the salivary CRP could not predict the serum CRP of the usually recommended cut-off > 1 mg/dL, with an AUC of 0.548 (95% CI: 0.43 to 0.66, *p*-value: 0.41). Due to the presence of heteroscedasticity in the generalized least squares method, the linear regression equation as per the Breusch Pagan test (BP = 23.056, df = 1, *p*-value < 0.001), a Box-Cox transformation was performed for the outcome variable, i.e., salivary CRP with a λ of 0.263. Upon Box-Cox transformation, homoscedasticity was achieved as per the Breusch Pagan test (BP = 0.774, df = 1, *p*-value = 0.379). The Pearson correlation coefficient between serum CRP and Box-Cox-transformed salivary CRP was moderate (r = 0.352, 95% CI: 0.135 to 0.537, *p*-value: 0.002) (Figure 4).

## 4. Discussion

In this cross-sectional analytical study, the bedside rapid salivary CRP estimations were found to be a good predictor of culture-positive sepsis in neonates with suspected sepsis. The novel rapid salivary CRP test by lateral flow method had almost similar sensitivity, specificity, NPV, PPV, and accuracy to serum CRP. Except in one report [20], all prior investigations [15,16,17,18,19] found significantly higher levels of salivary CRP in neonates with sepsis than those without sepsis. Salivary CRP has also been demonstrated to aid in diagnosing late-onset neonatal pneumonia [21]. Iyengar et al. [15] used an ELISA assay to assess the detection and potential utility of CRP in neonate saliva and discovered a statistically significant correlation between serum and salivary CRP (r = 0.62, *p* < 0.001), indicating that salivary CRP analysis is a viable screening tool for detecting abnormal serum CRP levels. Additionally, the investigators tried to adjust the salivary CRP values for the volume and protein concentration which were not needed in the lateral flow assay of the present study. A considerable and statistically significant positive connection between the salivary and serum CRP readings was found in a similar investigation by Datla et al. [17] using an indirect sandwich ELISA immunoassay kit in the entire study population (r = 0.63; *p* = 0.01). In the same study, the median salivary CRP levels were significantly different among neonates with culture-positive sepsis, screen-positive sepsis, and neonates with only risk factors for sepsis. Our research backs up these conclusions. Omran et al. [16] revealed a statistically significant difference in the mean salivary CRP between septic neonates and controls (12.0 ± 4.6 ng/L vs. 2.8 ± 1.2 ng/L), respectively, and at a cut-off point of 3.48 ng/L, with salivary C-reactive protein showing 94.3% sensitivity and 80% specificity, indicating a good predictive accuracy for predicting elevated serum C-reactive protein values in septic neonates. Similar to previous investigations, in our study, the correlation between salivary CRP and serum CRP levels was just moderate. Only very few studies evaluated the predictability of salivary CRP for sepsis and have found it to have good predictability, with varying AUC values of 0.886 and 0.63, respectively [16,22]. We found the predictability of salivary CRP for culture-positive sepsis in this study was very good with an AUC of 0.83. However, even when serum CRP levels were <1 mg/dL, higher salivary CRP values were continuously observed. The salivary CRP had AUC 95% CI < 0.5 in predicting serum CRP > 1 mg/dL, which was used as a cut-off for sepsis screening. These findings, together with a moderate correlation between serum and salivary CRP, suggest that salivary CRP values, despite having good AUC, sensitivity, specificity, PPV, and NPV in predicting culture-positive sepsis, are not always able to predict an increase in serum CRP levels. This could likely be explained by variations in salivary and serum CRP levels in different body matrices. More research is required to comprehend the up- and down-regulation of salivary CRP in an infected neonate through routine follow-ups and testing. In our study, the salivary CRP cut-off value was 11.6 ng/mL. The cut-off values are varied among the previous studies as most of the studies derived the cut-off scores for predicting serum CRP 10 mg/mL or more. In earlier studies, ELISA methods with different measuring units from ng/mL to mg/L were used. The time interval for salivary sample collection between the serum CRP was varied. In most of the studies, the investigators collected the salivary samples after 4–12 h of serum CRP. We performed salivary CRP testing within 1 h of serum CRP sampling. Most likely, this time variation and methodological differences led to the different cut-off values of salivary CRP and might also explain the different correlation values among the various studies. Nevertheless, further larger studies are needed to determine the optimal cut-off value of salivary CRP.

This was the first study to estimate salivary CRP using a rapid bedside test, obviating sample storage and transport. All the previous studies used ELISA for measuring the salivary CRP level, which required the preservation of the samples. Thawing and centrifugation were needed for the processing, which required training and laboratory support. Either a special swab [17] or a syringe with suction [15,16,20,21,22] or a device designed as a pacifier [13] were used to collect saliva samples. Large saliva samples were needed for this laborious collection procedure. A minimal volume (10 µL) of the salivary sample was collected using a sterile loop provided with the rapid test in our study. This method is very easy and causes less discomfort to neonates. In assessing if a sample is sufficient, the sample visual interpretation chart included in the quality control assessment kit will be very useful. As the test is rapid and does not need lab assistance, the turnaround time for the results is quite short.

The study’s strengths were using a rapid bedside device with a minimal salivary sample and analyzing the test to predict the clinically essential outcome, which was culture-positive sepsis. The significant limitations were being a small pilot study and not assessing the salivary CRP trend to understand CRP’s upregulation patterns and half-life in saliva.

Most of the organisms in our study were Gram-negative, so it needs to be tested in different organism profiles.

## 5. Conclusions

According to the study’s findings, the new rapid bedside tool for salivary CRP can be a potential non-invasive method in predicting culture-positive sepsis. Larger studies with adequately powered sample sizes are needed to corroborate the findings, define optimal cut-offs, and comprehend the up and down-regulation of salivary CRP in an infected neonate through routine follow-up.

## Figures and Tables

**Table 1:** Clinical characteristics of the study population.

Parameter	Variable (*n* = 74)
Gestational age (weeks) ^a^	34.1 ± 4.8
Birth weight (g) ^b^	2370 (1067–3182)
Male sex ^c^	44 (59.5)
Meconium-stained liquor ^c^	10 (13.5%)
PROM ^c^	23 (31)
Maternal urinary tract infection at delivery ^c^	3 (1.4)
Chorioamnionitis ^c^	14 (18.9)
Mode of delivery: Caesarean section ^c^	44 (59.5)
Required resuscitation ^c^	13 (17.6)
NICU admission ^c^	69 (93.2)
Leukopenia ^c^	8 (10.8)
Neutropenia ^c^	6 (8.1)
Received any respiratory support ^c^	64 (86)
CPAP/HFNC ^c^	49 (66)
Ventilation ^c^	15 (20)
Day of testing ^b^	1 (1–4)

^a^ = mean (±standard deviation), ^b^ = median (interquartile range), ^c^ = number (percentage); PROM: Premature rupture of membranes, NICU: neonatal intensive care unit.

**Table 2:** Serum and salivary CRP levels in three groups of neonates.

Test Variable	Blood Culture Positive Sepsis	Probable Sepsis	Neonates with Only Risk Factors	*p*-Value
Serum CRP (mg/dL) ^b^	3.87 (2–8.1)	1.8 (0.75–4)	0.1 (0.04–0.3)	<0.001
Salivary CRP	21 (7.9–38)	6.13 (2.2–12)	5.1 (1.3–12.5)	<0.001
(ng/mL) ^b^				

^b^ = median (interquartile range); CRP: C-reactive protein.

**Table 3:** Diagnostic characteristics of serum CRP and salivary CRP tests as predictors for blood culture positivity.

	Serum CRP	Salivary CRP
Area under the curve (AUC)	0.72 (0.58–0.86)	0.83 (0.70–0.97)
Optimal cut-off point in the study population	2.8 mg/dL	11.6 ng/mL
Sensitivity (%)	72 (46–90)	72 (46–90)
Specificity (%)	70 (56–81)	89 (78–96)
Positive predictive value (%)	43 (32–56)	68 (49–83)
Negative predictive value (%)	89 (78–94)	91 (83–95)
Accuracy (%)	70 (59–80)	85 (75–92)

CRP: C-reactive protein.

## Data Availability

The data will be shared by the corresponding author upon a reasonable request.

## Supplementary Materials

The following supporting information can be downloaded at: https://www.mdpi.com/article/10.3390/diagnostics13050867/s1, Figure S1: Chart illustrates different common test results and their visual interpretations for quality control assessment.
