# Supplementary material for: Examining the Utility of Rapid Salivary C-Reactive Protein as a Predictor for Neonatal Sepsis: An Analytical Cross-Sectional Pilot Study
Source: Diagnostics (Basel). 2023 Feb 24;13(5):867. doi: 10.3390/diagnostics13050867 (PMC10000952; doi:10.3390/diagnostics13050867)
Supplement: Supplementary file 1 [file diagnostics-13-00867-s001.zip › diagnostics-2146552-supplementary.pdf]

Supplementary material Figure S1: Chart illustrates different common test results and their visual interpretations for quality control assessment

Visual interpretations chart:

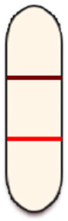	**Sharp control and test lines:** Test has run appropriately and the biomarker has been identified in the sample. The cartridge can be inserted in VIEWDx to quantify the level and further analyze the results.
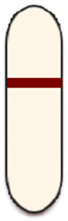	**Sharp control line but no test line:** Test has run appropriately but the level of biomarker is below the limit of detection. This cartridge can also be inserted in VIEWDx for generation of results and also archiving the data.
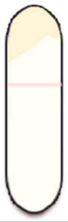	**Incomplete run of test (Err:01):** In case the test strip looks like the cartridge in the figure; the test has dried mid-run. This happens when the sample volume was inappropriate or sample is too viscous and cannot flow properly on the membrane. In such cases, the test should be run again.
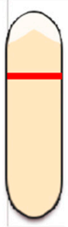	**Very high background (Err:02):** In case the test strip looks like the cartridge in the figure; there is an extreme high background due to low sample release. This can be resolved by waiting for an additional minute or adding an extra drop of assay diluent at the sample well. If the high red background color doesn't clear after these measures, the test should be redone.
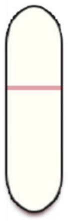	**Extremely faint control line (Err:03):** In case the test strip looks like the cartridge in the figure; the test hasn't run properly and should be redone.
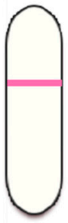	**Extremely faint test line (which vanishes after couple of minutes) (Err:04)** In case the test strip looks like the cartridge in the figure; the sample has very low biomarker levels. In such cases it is advisable to repeat the test again after some time to eliminate any false negative results.
